# Effectiveness of Organizational Interventions to Reduce Emergency Department Utilization: A Systematic Review

**DOI:** 10.1371/journal.pone.0035903

**Published:** 2012-05-02

**Authors:** Gemma Flores-Mateo, Concepción Violan-Fors, Paloma Carrillo-Santisteve, Salvador Peiró, Josep-Maria Argimon

**Affiliations:** 1 Institut Universitari d'Investigació en Atenció Primària Jordi Gol, Barcelona, Spain; 2 Universitat Rovira i Virgili, Tarragona, Spain; 3 Universitat Autònoma de Barcelona, Bellaterra, Spain; 4 Institut Català de la Salut, Catalunya, Spain; 5 Centro Superior de Investigación en Salud Pública, Valencia, Spain; 6 Divisió d'Avaluació, Servei Català de la Salut, Barcelona, Spain; Yale University School of Medicine, United States of America

## Abstract

**Background:**

Emergency department (ED) utilization has dramatically increased in developed countries over the last twenty years. Because it has been associated with adverse outcomes, increased costs, and an overload on the hospital organization, several policies have tried to curb this growing trend. The aim of this study is to systematically review the effectiveness of organizational interventions designed to reduce ED utilization.

**Methodology/Principal Findings:**

We conducted electronic searches using free text and Medical Subject Headings on PubMed and The Cochrane Library to identify studies of ED visits, re-visits and mortality. We performed complementary searches of grey literature, manual searches and direct contacts with experts. We included studies that investigated the effectiveness of interventions designed to reduce ED visits and the following study designs: time series, cross-sectional, repeated cross-sectional, longitudinal, quasi-experimental studies, and randomized trial. We excluded studies on specific conditions, children and with no relevant outcomes (ED visits, re-visits or adverse events). From 2,348 potentially useful references, 48 satisfied the inclusion criteria. We classified the interventions in mutually exclusive categories: 1) Interventions addressing the supply and accessibility of services: 25 studies examined efforts to increase primary care physicians, centers, or hours of service; 2) Interventions addressing the demand for services: 6 studies examined educational interventions and 17 examined barrier interventions (gatekeeping or cost).

**Conclusions/Significance:**

The evidence suggests that interventions aimed at increasing primary care accessibility and ED cost-sharing are effective in reducing ED use. However, the rest of the interventions aimed at decreasing ED utilization showed contradictory results. Changes in health care policies require rigorous evaluation before being implemented since these can have a high impact on individual health and use of health care resources. Systematic review registration: http://www.crd.york.ac.uk/PROSPERO. Identifier: *CRD420111253*

## Introduction

The use of emergency departments (ED) has increased dramatically in developed countries, with a large portion of this increase attributed to inappropriate or non-urgent visits [1], [2]. The healthcare community, as well as the society at large, is concerned about ED overuse for reasons beyond the higher costs compared to Primary Care. When patients replace Primary Care with ED visits there is a lack of continuity and follow-up that limits the provider's awareness of previous and current illness and treatments and makes it difficult to engage in shared decision making; ED resources may be diverted from life-threatening situations to minor health problems; and the ED requests for “urgent” tests and explorations may generate an overload that can adversely affect the hospital as a whole. Finally, ED overuse can be a source of staff frustration and patient dissatisfaction [3], [4].

Many studies have examined the circumstances which may contribute to increasing ED visits [5]–[7]. Reasons proposed to explain the trend towards an increasing number of patients using the ED as a type of primary care include the progressive aging of the population and the associated increase in chronic conditions, lack of cost awareness, organizational problems in primary care, better ED convenience and accessibility, and patients' subjective perception of illness severity and greater confidence in the ED compared to primary care services [7].

Several interventions have been developed to decrease the utilization of ED services, from healthcare education to measures that limit access to ED (e.g., mandatory gatekeeping, co-payment) or improve the accessibility of primary care or alternative services (such as urgent care outside of normal office hours).

Recently, a systematic review aimed to assess the type and effectiveness of interventions to reduce the number of ED visits by frequent users has been published [8]. However, frequent users represent about a quarter (21% to 28% [9]) of all ED visits and are not the only reason for growth in ED use. Our aim is to systematically review the effectiveness of organizational interventions intended to reduce ED utilization in the general population.

## Methods

### 1) Study design

Systematic review of studies that investigated ED visits, and specifically to determine the effectiveness of interventions designed to reduce ED visits.

### 2) Search strategy and study selection

A search strategy was developed to identify relevant studies, and was adapted for each database searched (see Table S1 for details of terms used in the PubMed and The Cochrane Library search). In addition to “emergency medical services” and “emergency department”, in order to capture a broad range of outcomes associated with ED effectiveness and safety we included the following search terms: cost-benefit analysis, cost-effectiveness evaluation, effectiveness, utilization, efficacy, health care quality, access, length of stay, waiting time, costs, health services and accessibility. To identify organizational interventions we included search terms identified through early scoping searches: health education, patient education, out-of-hours, after-hours, walk-in centers, continuing care, fast track areas, fast track unit, nurse practitioners, nurse manager, triage, hotline, helpline, telephone consultation, telephone triage, copayment, cost sharing, incentive based, coinsurance, tiered benefit, patient charge, gatekeeping and primary health care.

The search period was January 1985 to February 2012. There were no language restrictions. We also searched the references included in published reviews [10]–[14] and one unpublished working paper [15]. We also contacted various experts asking for additional primary studies (particularly unpublished or recently published studies).

Three researchers (GFM, PCS and CVF) reviewed the titles and abstracts of all studies identified. Cases of discordance were resolved by consensus; as necessary, the entire article was read to select relevant studies on the effect of any type of organizational interventions on ED utilization.

Since the majority of these studies analyzed observational data, understanding how the association between intervention and the outcomes of interest were measured is important. Primary outcome was ED visits and the secondary outcomes were re-visits, hospital admissions, mortality or safety measures.

We included the following study designs: a) Time series, an analysis of changes over time in data aggregated at the geographic or plan level, with the data spanning a period when benefits changed; b) Cross-sectional, an analysis of individual-level data at a single time point; c) Repeated cross-sectional, an analysis of cross-sectional data from multiple time periods; d) Longitudinal, an analysis of individual-level data with repeated observations for the same beneficiaries over time; e) Quasi-experimental studies, which compared outcomes at two points in time, before and after a benefit change; f) Randomized trial.

We excluded studies that targeted only specific conditions such as diabetes, asthma, or cardiac failure in an effort to increase homogeneity and comparability between studies, and because patients with specific conditions showed different patterns of ED visitation with higher hospital re-admission. We also excluded studies with no relevant outcomes (ED visits, ED re-visits or safety measures), and non-original studies. Our analysis was limited to adults (age ≥18 years) presenting to ED because, in several countries, patterns of utilization in pediatric services are markedly different from those of adults [16], [17].

When several articles using the same population were published, the publication with the longest follow-up was selected.

### 3) Data extraction and quality assessment

Relevant information from selected articles was extracted by three researchers (GFM, PCS and CVF) and summarized in several ways (**Tables S2, S3 and S4**). All selected articles were reviewed by three reviewers, achieving 96% inter-rater agreement. Discrepancies were resolved by consensus. We used a standardized data-collection form to record publication year, country, design, participants, sample size, outcome, intervention, duration and frequency of intervention, and instruments for evaluating the intervention effectiveness. The primary outcome was ED use and the secondary outcomes were ED re-visits and mortality.

To assess the methodological quality of the studies, two authors (GFM and CVF) used seven binary criteria based on those used in previous systematic reviews and applicable across the range of study designs included in the present analysis [18]. Each positive answer was scored as 1, and negative findings were scored as 0.The overall score ranged from 0 to 7 based on the following seven criteria:

The seven criteria were: a) Randomization of participants, groups or areas to intervention or control groups; b) Exposure, when the authors show that participants and control groups did not receive concurrent interventions that could have differentially influenced the studied interventions, and that control groups were not contaminated by having received some or all of an intervention being studied; c) Representativeness of the study population, achieving a recruitment response rate of at least 60%, or when participants were shown to be a representative sample of the study population; d) Comparability of baseline characteristics of intervention and control groups, populations or areas; or if important baseline differences in potential confounders did exist, the data analysis had been adjusted appropriately; e) Attrition or sample size, with a standard of less than 30% in a cohort or panel of respondents or, in a repeated cross-sectional design, analysis of a minimum sample of 100 participants in each wave of intervention as well as control groups; f) Period of outcomes assessment longer than 6 months; g) Instruments used to assess interventions, i.e., appropriate for the purpose of measuring the outcomes under consideration and shown to be a valid and reliable measure in published research, or in a pilot study. Disagreement between reviewers was resolved by consensus.

### 4) Evidence synthesis

We analyzed the existing organizational interventions, which were included from the perspective of the services on supply or the demand made on these services.

The interventions addressing the supply of services were: 1) Interventions to improve accessibility to primary care, with three subcategories: 1.1) Interventions that improve non-ED primary care access by gross supply (increasing number of primary care centers or primary care physicians); 1.2) Interventions that improve productivity (e.g., increasing hours of access to medical services). In this second subcategory we identified different models such as practice-based services (General Practitioners within a practice looking after their own patients after hours), deputizing services (commercial companies employing doctors to provide after-hours service), extra-hospital emergency departments (primary care patients using non-hospital emergency department out-of-hours), cooperatives (General Practitioners from different practices forming a non-profit organization to provide care for their own patients out-of-hours); and 1.3) Telephone triage and advice services (the use of telephone consultation for primary care patients seeking medical help out-of-hours)

The interventions addressing the demand for services were: 2) Educational interventions, included only if the intervention was not accompanied by non-educational components; 3) Barrier interventions such as gatekeeping (defined as patients who do not have direct access to secondary care and need a referral from a general practitioner or health maintenance organization to get access to ED [19]), and cost-sharing (defined as any kind of out-of pocket payment for health care services). We included the most widely used forms of cost-sharing: co-payments (patients pay a flat fee for each medical service sought or product purchased), coinsurance (patients pay a fixed percentage of the cost of care), and deductibles (the amount one must pay out of pocket annually before insurance coverage begins to pay) [20].

The interventions, study designs, participants, outcomes and duration of follow-up were too heterogeneous to support quantitative pooling. Thus, the conclusions of this review are necessarily qualitative.

Systematic review registration: http://www.crd.york.ac.uk/PROSPERO. Identifier: *CRD420111253*

## Results

**Figure 1:**
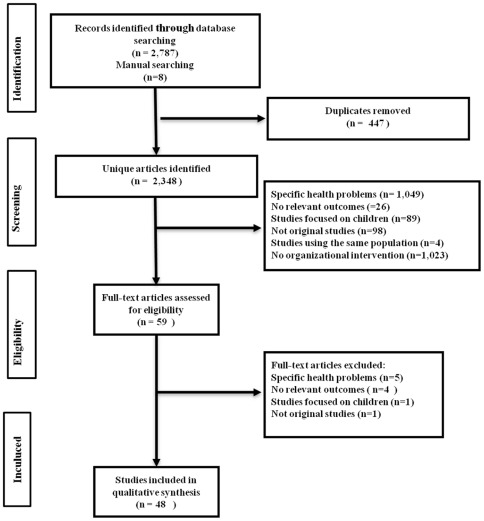
Flow of articles through the literature review process.

Of 2,348 screened studies, 48 satisfied the inclusion criteria (Figure 1). After independent review of all references, the rate of agreement between reviewers of the 2,348 total references was 86% and lack of agreement was resolved by consensus.

### 1) Interventions that address the supply of services

#### Interventions aimed to improve primary care accessibility

Twenty-five studies [2], [21]–[44] examined interventions to improve accessibility to primary care.

Ten studies [21]–[30] were focused in interventions that increased primary care medical doctors or primary care centers. One study was a randomized controlled trials [25], three were quasi-experimental studies with control group [21], [22], [26], one was a quasi-experimental study without control group [30], one was a case-control study [29], and the rest were cross-sectional [23], [24], [27], [28]. Five studies were from the US [22], [25], [26], [29], [30], and the rest from Canada [28], Spain [23], Sweden [21] and Brazil [27]. (**Table S2**).

The evidence clearly demonstrates that increased numbers of primary care centers or medical doctors is associated with lower ED visits. In a quasi-experimental study with a control group, Sjonell et al. found that after a primary care center was established, visits to ED were reduced by 40% [21]. More recently, Retchin et al. found that using community primary care physicians to coordinate care for the uninsured seems to reduce emergency department use (73.9% vs 42.9%; P<0.001) [30]. Also, individuals who had a primary care physician were more likely to present to the ED appropriately [28]. Most of the evidence on this point comes from quasi-experimental studies [21], [22], [26] of Medicaid programs or uninsured individuals [30]. In relation to the person providing the care, one randomized controlled trial in which 209 patients were randomized to a General Practitioner or to a hospital internist observed a lower number of ED visits in patients assigned to General Practitioners, compared to patients assigned to the hospital internist (effect size −0.204 (95% confidence interval −0.378 to −0.029) [25].

Three studies have examined changes in hospital admissions [25], [26], [30], two studies found a decrease in hospital admissions [26], [30], and one study did not find significant differences in charges for hospitalization [25]. None of the studies examined mortality.

Evidence from nine studies [2], [31]–[38] examined the association between out-of-hours services and ED utilization (**Table S2**): three quasi-experimental studies with control groups [35], [36], [38]; two quasi-experimental studies without control groups [31], [34]; two time-series studies [2], [33]; one cohort study [37]; and one cross-sectional study [32].

Three studies were from Spain [2], [31], [32], and three were from UK [33], [35], [38]. The rest of the studies were from the US [37], the Netherlands [34] and Belgium [36].

Two quasi-experimental studies found a decrease of ED utilization after increasing hours of primary care access [31], [34]. Valdres et al. found that after installation of extra-hospital emergency services, daily average of ED visits fell significantly (effect sized −0.38 (confidence interval 95% −0.56 to −0.20) [31]. Moreover, Van Uden et al. found visits to ED were reduced 53% after the establishment of a primary care physician cooperative [34].

A quasi-experimental study with a control group found that after installation of a walk-in center, the ED attendance rate increased by 10%. Furthermore, a time series study showed a mean increase of 7.8% in the number of emergencies in continuing care points and a mean increase of 5.1% in ED [2]. When the different types of extra-hospital emergency services were segregated and compared on the basis of who provided the care and where the services were located, the data showed that visits to the ED were reduced when the attention was provided by the same primary care team [32]. Only one study assessed mortality [34]. No changes in mortality were found.

Six studies [39]–[44] evaluated the effect of telephone triage and consultation (four of which were randomized controlled trials). None of these found significant differences in the number of ED attendance incidents between groups. Most of the studies were performed in the UK [40]–[44]. Two studies [42], [43] showed an increase in re-visits for the same condition.

### 2) Interventions that address the demand for services

#### Educational interventions

Evidence from six studies [45]–[50] that examined the effect of educational interventions on the utilization of ED services and safety outcomes is summarized in **Table S3**. There were three randomized controlled trials [45], [47], [48], one quasi-experimental study with a control group [49], one quasi-experimental study without a control group [50], and one case-control study [46]. Two studies were from Australia [47], [49], and the rest of the studies were from the US [45], [46], [48], [50].

The randomized controlled trials with large sample sizes and high quality [45], [47] did not observe differences in ED utilization following the educational interventions. However, Scott et al. performed a randomized controlled trial which found that monthly group meetings with educational components had fewer emergency visits than the control group (effect size −0.313 (CI95% −0.543, −0.003) [48]. A quasi-experimental study that evaluated three interventions (Health education, teaching patients how to use the health care system and providing counselling in social/emotional issues) was significantly correlated with a decrease in ED visits [50]. A quasi-experimental study in which participants were allocated a “care facilitator” who provided assistance in identifying and accessing required health care services, as well as education in aspects of self management, found a 20.8% reduction in ED visits [49]. However, this reduction disappeared at the three-month follow-up in a case-control study [46].

Two articles assess mortality rates [47], [48] and hospital admissions [49]. Neither showed differences in mortality rates between the intervention and control group. In addition, the studies consistently found a significant reduction of hospital admissions in the intervention groups [47]–[49].

#### Barrier interventions

Evidence from 12 studies [51]–[62] examined the association between ED cost-sharing and ED visits and safety outcomes (**Table S4**). The Health Insurance Experiment randomized 2,750 families to different levels of cost-sharing ranging from free care to 90% coinsurance [51]. The absence of cost-sharing resulted in significantly greater ED use than insurance with cost-sharing. However, the Health Insurance Experiment also found that cost-sharing reduced both non-urgent and urgent visits [51]. No other randomized studies have been performed.

Six quasi-experimental studies with control group [57]–[62] and one quasi-experimental study without control group [53] have examined the impact of ED cost-sharing on ED utilization. All but one of these studies found that ED cost-sharing reduces ED utilization. One study found a dose-response relationship between co-payment and ED visits reduction [57]. Wharam et al. [58] and Wilson et al. [59] tracked people during their first year of enrollment in a health plan with high deductibles, and ED use fell in both studies.

Two cross-sectional studies examined perceived levels of copayments for ED utilization and measured how cost-sharing affected patients' decisions about where or when to seek care [55], [56].On average, 41% of the subjects correctly reported the amount of the copayment, and 9% delayed going to the ED [56].

Consistent with the findings that ED cost-sharing reduces ED visits, the studies showed no increases in hospitalizations or mortality rates.

Five studies, all performed in the US [63]–[67], evaluated the effect of gatekeeping on ED utilization and safety (**Table S4**). The studies evaluated the effect of the Health Maintenance Organization (HMO) in analyzing the effect of “primary care management” as a specific model of managed care. This required a pre-authorization of payment for the ED visit via the “managed care gate-keeper”. Only one randomized controlled trial [66], in which the usual primary care doctor carried out the gatekeeping role, showed no increases in hospital emergencies or mortality rates. One cross-sectional study found that gatekeeping plans were successful in reducing ED use for enrolees as contrasted with the control group [67]. Those studies that analyzed recurrent visits and adverse effects observed re-visits rates of between 11% [65] and 24.1% [64].

### 3) Study quality

The coefficient of correlation of reviewer agreement in evaluating the quality of the studies was 0.72 (P<0.001). Quality and methodological reporting were poor in most of the studies (**Tables S2 to S4**), showing a median value of 3 (range: 2–4). There were large variations in study design and, although randomized controlled trials had a tendency to fulfil quality criteria while the rest of studies varied widely, only a few studies had randomized controlled trial design.

## Discussion

The systematic review and qualitative evaluations of the literature indicate that interventions aimed to increase primary care accessibility such as increasing primary care medical doctors, primary care centers and cost-sharing are effective in reducing ED visitation. The remaining interventions showed contradictory results.

Our review found consistent evidence that ED cost-sharing successfully reduces ED utilization. Apparently, people who should go to the ED are not deterred by co-payments, whereas at least some of those who should not be using the ED are deterred [20]. However, the impact of cost-sharing in different subgroups is limited. Notably absent are studies that assess the effect of cost-sharing in populations with low purchasing power and in the more disadvantaged social classes which, in general, are those that most frequently utilize hospital ED facilities [68].

Overall, studies that focused on interventions aimed at increasing out-of-hours primary care services did not showed a reduction in ED visits, although these studies received low global quality ratings and various different models of out-of-hours primary medical care services were identified. Most of these studies were performed in countries with a National Health System that includes strong primary health care. The studies showed that ED visits fell after the installation of extra-hospital emergency services [31]. However, the long-term effect is the increasing percentage of patients entering the health care system through ED, rather than through extra-hospital emergency services [2], [38].

Interventions aimed to improve primary care accessibility was associated with a decrease in ED visits. Patients who have an ongoing health care relationship with their family physician are more likely to seek the opinion of their physician before soliciting assistance from the ED, especially when the urgency of the attention sought may be in doubt [69].

The evidence showed that telephone consultation interventions were associated with an increase in the number of re-visits for the same health problem; this system, in reality, delays the visit rather than resolving the problem [42], [43].

We found that interventions directed towards demand, such as educational interventions, were not effective in reducing ED utilization when the intervention was stand-alone, i.e. the intention being merely to educate patients regarding overall health service utilization. However, educational interventions are effective in reducing hospital admissions. It is interesting to compare these results with previous systematic reviews focused on specific chronic problems [70], [71]. These systematic reviews showed that educational interventions seem more effective when they are introduced as a part of a multi-faceted intervention, or even in the treatment of specific chronic conditions [70], [71].

The studies aimed at filtering access to ED, such as gatekeeping, found little impact on ED visits. This is consistent with studies that assess the effect of gatekeeping on use of health services such as visits to specialists [72], [73]. We identified two different gatekeeper plans. In the first, used in the UK and Scandinavian countries, GPs have a gatekeeping role in the health care system. Patients do not have direct access to secondary care; they need a referral from their GP to get access to a hospital. In the second gatekeeping plan, used in the US, health maintenance organizations practice gatekeeping, but with no standardized triage criteria and with various personnel functioning as the gatekeeper. The studies identified and included in our systematic review analyze the second type of gatekeeping, and only one study used a randomized controlled design.

Our study has several limitations. While the bias of identification and selection are a possible threat to validity in all systematic reviews, this problem is accentuated when non-randomized studies are included. Non-randomized studies are more difficult to identify than those that are randomized because they involve greater variation in the design, and there is no standardized terminology or keywords. The inclusion of all relevant studies in systematic reviews is crucial to avoid bias and maximize precision; for this reason we supplemented searches of databases with several sources such as review references from published and unpublished papers, consultations with experts and we included studies published in languages other than English. However, we cannot exclude the bias completely.

Another limitation was that the majority of articles examined were outcomes studies conducted using administrative data and did not control for potential confounding variables (socio-economic status, comorbidity, age, sex, etc.). As such, the results must be interpreted with caution.

Finally, we find a high degree of heterogeneity of acute care system delivery across developed countries. Interventions may be geographically sensitive, and therefore our findings may be limited. For example, the patient's ability to pay for access to emergency health services has been studied mainly in countries where health care provision is covered by private insurance such as the US. The component of access is different from that of the State-funded universal health coverage systems employed in most other developed countries. The effective utilization of emergency services while preventing overload is a complex and multi-factorial problem that requires integrated interventions with respect to the organization of, and benefit from, emergency services. These interventions would be specific for each country and would need to be implemented as a function of the coverage and funding of the individual country's health-care system.

Our review also has several strengths. The questions addressed are timely and of major importance in health policy decisions. Dramatic increases in ED utilization require that equitable solutions be sought and, having included several studies published in languages other than English, the current report provides us with an insight into how the problem is addressed in different countries.

Despite the limitations of the present systematic review, the information available is sufficient to establish specific guidelines for clinical practice and to highlight future research in this area.

Several research issues remain unresolved and require future research. First, with respect to the safety of interventions, most studies do not measure patients' health, morbidity or even mortality. In our opinion, safety is an essential point before implementing such new policies. As with the US Food and Drug Administration (FDA), which evaluates safety and efficacy before new drugs are approved for use, health policy changes also must be evaluated thoroughly before implementation, since such changes in policy can do “more harm than good” [74].

Assessing the potential effect of organizational interventions on decreased ED utilization is complicated by the subjectivity in the interpretation of results due to the high number of interventions evaluated, as well as the manner of results/outcomes measurement and the different populations or acute care system delivery systems in developed countries. These variations make comparison between studies difficult and meta-analysis almost impossible.

In sum, the evidence suggests that interventions aimed at increasing primary care accessibility and ED cost-sharing are effective in reducing ED use. However, the rest of the interventions aimed at decreasing ED utilization showed contradictory results. Changes in health care policies require rigorous evaluation before being implemented since these can have a high impact on individual health and use of health care resources.

## Supporting Information

Table S1 **Search strategy used for systematic review.**
(DOC)

Table S2 **Studies examining interventions to improve accessibility of Primary Care (PC).**
(DOC)

Table S3 **Studies examining educative intervention.**
(DOC)

Table S4 **Studies examining barriers interventions.**
(DOC)
