# Supplementary material for: Effectiveness of Organizational Interventions to Reduce Emergency Department Utilization: A Systematic Review
Source: PLoS One. 2012 May 2;7(5):e35903. doi: 10.1371/journal.pone.0035903 (PMC3342316; doi:10.1371/journal.pone.0035903)
Supplement: Table S1 — Search strategy used for systematic review. (DOC) [file pone.0035903.s001.doc]

**Table S1.** Search strategy used for systematic review.

| **PUBMED** | | | | |
| --- | --- | --- | --- | --- |
| **Search** | **Query** | | **Items found** | |
| [#1](http://www.ncbi.nlm.nih.gov/pubmed/advanced) | “Health education” | | [68209](http://www.ncbi.nlm.nih.gov/pubmed/?cmd=HistorySearch&querykey=1) | |
| [#2](http://www.ncbi.nlm.nih.gov/pubmed/advanced) | “patient education” | | [70867](http://www.ncbi.nlm.nih.gov/pubmed/?cmd=HistorySearch&querykey=2) | |
| [#3](http://www.ncbi.nlm.nih.gov/pubmed/advanced) | “out-of-hours” | | [887](http://www.ncbi.nlm.nih.gov/pubmed/?cmd=HistorySearch&querykey=3) | |
| [#4](http://www.ncbi.nlm.nih.gov/pubmed/advanced) | “walk-in centers” | | [8](http://www.ncbi.nlm.nih.gov/pubmed/?cmd=HistorySearch&querykey=4) | |
| [#5](http://www.ncbi.nlm.nih.gov/pubmed/advanced) | “continuing care” | | [1425](http://www.ncbi.nlm.nih.gov/pubmed/?cmd=HistorySearch&querykey=5) | |
| [#6](http://www.ncbi.nlm.nih.gov/pubmed/advanced) | “fast track areas” | | [9](http://www.ncbi.nlm.nih.gov/pubmed/?cmd=HistorySearch&querykey=6) | |
| [#7](http://www.ncbi.nlm.nih.gov/pubmed/advanced) | “fast track unit” | | [3](http://www.ncbi.nlm.nih.gov/pubmed/?cmd=HistorySearch&querykey=7) | |
| [#8](http://www.ncbi.nlm.nih.gov/pubmed/advanced) | “nurse practitioners” | | [15581](http://www.ncbi.nlm.nih.gov/pubmed/?cmd=HistorySearch&querykey=8) | |
| [#9](http://www.ncbi.nlm.nih.gov/pubmed/advanced) | “nurse manager” | | [625](http://www.ncbi.nlm.nih.gov/pubmed/?cmd=HistorySearch&querykey=9) | |
| [#10](http://www.ncbi.nlm.nih.gov/pubmed/advanced) | “triage” | | [11138](http://www.ncbi.nlm.nih.gov/pubmed/?cmd=HistorySearch&querykey=10) | |
| [#11](http://www.ncbi.nlm.nih.gov/pubmed/advanced) | “hotline” | | [679](http://www.ncbi.nlm.nih.gov/pubmed/?cmd=HistorySearch&querykey=11) | |
| [#12](http://www.ncbi.nlm.nih.gov/pubmed/advanced) | “helpline” | | [285](http://www.ncbi.nlm.nih.gov/pubmed/?cmd=HistorySearch&querykey=12) | |
| [#13](http://www.ncbi.nlm.nih.gov/pubmed/advanced) | ”telephone consultation” | | [240](http://www.ncbi.nlm.nih.gov/pubmed/?cmd=HistorySearch&querykey=13) | |
| [#14](http://www.ncbi.nlm.nih.gov/pubmed/advanced) | “ telephone triage” | | [281](http://www.ncbi.nlm.nih.gov/pubmed/?cmd=HistorySearch&querykey=14) | |
| [#15](http://www.ncbi.nlm.nih.gov/pubmed/advanced) | “copayment” | | [356](http://www.ncbi.nlm.nih.gov/pubmed/?cmd=HistorySearch&querykey=15) | |
| [#16](http://www.ncbi.nlm.nih.gov/pubmed/advanced) | "cost sharing" | | [2229](http://www.ncbi.nlm.nih.gov/pubmed/?cmd=HistorySearch&querykey=16) | |
| [#17](http://www.ncbi.nlm.nih.gov/pubmed/advanced) | "incentive based" | | [155](http://www.ncbi.nlm.nih.gov/pubmed/?cmd=HistorySearch&querykey=17) | |
| [#18](http://www.ncbi.nlm.nih.gov/pubmed/advanced) | "coinsurance" | | [1438](http://www.ncbi.nlm.nih.gov/pubmed/?cmd=HistorySearch&querykey=18) | |
| [#19](http://www.ncbi.nlm.nih.gov/pubmed/advanced) | "tiered benefit" | | [1](http://www.ncbi.nlm.nih.gov/pubmed/?cmd=HistorySearch&querykey=19) | |
| [#20](http://www.ncbi.nlm.nih.gov/pubmed/advanced) | "patient charge" | | [55](http://www.ncbi.nlm.nih.gov/pubmed/?cmd=HistorySearch&querykey=20) | |
| [#21](http://www.ncbi.nlm.nih.gov/pubmed/advanced) | "gatekeeping" | | [749](http://www.ncbi.nlm.nih.gov/pubmed/?cmd=HistorySearch&querykey=21) | |
| [#22](http://www.ncbi.nlm.nih.gov/pubmed/advanced) | "primary health care" | | [56601](http://www.ncbi.nlm.nih.gov/pubmed/?cmd=HistorySearch&querykey=22) | |
| [#23](http://www.ncbi.nlm.nih.gov/pubmed/advanced) | (#1 OR #2 OR #3 OR #4 OR #5 OR #6 OR #7 OR #8 OR #9 OR #10 OR #11 OR #12 OR #13 OR #14 OR #15 OR #16 OR #17 OR #18 OR #19 OR #20 OR #21 OR #22) | | [218772](http://www.ncbi.nlm.nih.gov/pubmed/?cmd=HistorySearch&querykey=23) | |
| [#24](http://www.ncbi.nlm.nih.gov/pubmed/advanced) | "emergency medical services" | | [30529](http://www.ncbi.nlm.nih.gov/pubmed/?cmd=HistorySearch&querykey=24) | |
| [#25](http://www.ncbi.nlm.nih.gov/pubmed/advanced) | "emergency department" | | [36790](http://www.ncbi.nlm.nih.gov/pubmed/?cmd=HistorySearch&querykey=25) | |
| [#26](http://www.ncbi.nlm.nih.gov/pubmed/advanced) | (#24 OR #25) | | [64547](http://www.ncbi.nlm.nih.gov/pubmed/?cmd=HistorySearch&querykey=26) | |
| [#27](http://www.ncbi.nlm.nih.gov/pubmed/advanced) | "Cost-benefit analysis" | | [53407](http://www.ncbi.nlm.nih.gov/pubmed/?cmd=HistorySearch&querykey=27) | |
| [#28](http://www.ncbi.nlm.nih.gov/pubmed/advanced) | "effectiveness" | | [228295](http://www.ncbi.nlm.nih.gov/pubmed/?cmd=HistorySearch&querykey=28) | |
| [#29](http://www.ncbi.nlm.nih.gov/pubmed/advanced) | "utilization” | | [225628](http://www.ncbi.nlm.nih.gov/pubmed/?cmd=HistorySearch&querykey=29) | |
| [#30](http://www.ncbi.nlm.nih.gov/pubmed/advanced) | ”efficacy” | | [411970](http://www.ncbi.nlm.nih.gov/pubmed/?cmd=HistorySearch&querykey=30) | |
| [#31](http://www.ncbi.nlm.nih.gov/pubmed/advanced) | ”health care quality” | | [1602](http://www.ncbi.nlm.nih.gov/pubmed/?cmd=HistorySearch&querykey=31) | |
| [#32](http://www.ncbi.nlm.nih.gov/pubmed/advanced) | “access” | | [145977](http://www.ncbi.nlm.nih.gov/pubmed/?cmd=HistorySearch&querykey=32) | |
| [#33](http://www.ncbi.nlm.nih.gov/pubmed/advanced) | “length of stay” | | [59382](http://www.ncbi.nlm.nih.gov/pubmed/?cmd=HistorySearch&querykey=33) | |
| [#34](http://www.ncbi.nlm.nih.gov/pubmed/advanced) | ” waiting time” | | [3630](http://www.ncbi.nlm.nih.gov/pubmed/?cmd=HistorySearch&querykey=34) | |
| [#35](http://www.ncbi.nlm.nih.gov/pubmed/advanced) | “costs" | | [155450](http://www.ncbi.nlm.nih.gov/pubmed/?cmd=HistorySearch&querykey=35) | |
| [#36](http://www.ncbi.nlm.nih.gov/pubmed/advanced) | "Health Services Accessibility” | | [42175](http://www.ncbi.nlm.nih.gov/pubmed/?cmd=HistorySearch&querykey=36) | |
| [#37](http://www.ncbi.nlm.nih.gov/pubmed/advanced) | (#27 OR #28 OR #29 OR #30 OR #31 OR #32 OR #33 OR #34 OR #35 OR #36) | | [1151828](http://www.ncbi.nlm.nih.gov/pubmed/?cmd=HistorySearch&querykey=37) | |
| [#38](http://www.ncbi.nlm.nih.gov/pubmed/advanced) | (#23 AND #26 AND #37) | | [2361](http://www.ncbi.nlm.nih.gov/pubmed/?cmd=HistorySearch&querykey=38) | |
| **THE COCHRANE LIBRARY** | | | | |
| **Search** | | **Query** | | **Items found** |
| #1 | | ["Health education"](http://onlinelibrary.wiley.com/o/cochrane/searchHistory?mode=runquery&qnum=1) | | 5067 |
| #2 | | ["patient education"](http://onlinelibrary.wiley.com/o/cochrane/searchHistory?mode=runquery&qnum=2) | | 7006 |
| #3 | | ["out-of-hours"](http://onlinelibrary.wiley.com/o/cochrane/searchHistory?mode=runquery&qnum=3) | | 79 |
| #4 | | ["walk-in centers"](http://onlinelibrary.wiley.com/o/cochrane/searchHistory?mode=runquery&qnum=4) | | 0 |
| #5 | | ["continuing care"](http://onlinelibrary.wiley.com/o/cochrane/searchHistory?mode=runquery&qnum=5) | | 142 |
| #6 | | ["fast track areas"](http://onlinelibrary.wiley.com/o/cochrane/searchHistory?mode=runquery&qnum=6) | | 2 |
| #7 | | ["fast track unit"](http://onlinelibrary.wiley.com/o/cochrane/searchHistory?mode=runquery&qnum=7) | | 0 |
| #8 | | ["nurse practitioners"](http://onlinelibrary.wiley.com/o/cochrane/searchHistory?mode=runquery&qnum=8) | | 518 |
| #9 | | ["nurse manager"](http://onlinelibrary.wiley.com/o/cochrane/searchHistory?mode=runquery&qnum=9) | | 22 |
| #10 | | ["triage"](http://onlinelibrary.wiley.com/o/cochrane/searchHistory?mode=runquery&qnum=10) | | 608 |
| #11 | | ["hotline"](http://onlinelibrary.wiley.com/o/cochrane/searchHistory?mode=runquery&qnum=11) | | 100 |
| #12 | | ["helpline"](http://onlinelibrary.wiley.com/o/cochrane/searchHistory?mode=runquery&qnum=12) | | 58 |
| #13 | | ["telephone consultation"](http://onlinelibrary.wiley.com/o/cochrane/searchHistory?mode=runquery&qnum=13) | | 72 |
| #14 | | ["telephone triage"](http://onlinelibrary.wiley.com/o/cochrane/searchHistory?mode=runquery&qnum=14) | | 43 |
| #15 | | ["copayment"](http://onlinelibrary.wiley.com/o/cochrane/searchHistory?mode=runquery&qnum=15) | | 61 |
| #16 | | ["cost sharing"](http://onlinelibrary.wiley.com/o/cochrane/searchHistory?mode=runquery&qnum=16) | | 87 |
| #17 | | ["incentive based"](http://onlinelibrary.wiley.com/o/cochrane/searchHistory?mode=runquery&qnum=17) | | 26 |
| #18 | | ["coinsurance"](http://onlinelibrary.wiley.com/o/cochrane/searchHistory?mode=runquery&qnum=18) | | 45 |
| #19 | | ["tiered benefit"](http://onlinelibrary.wiley.com/o/cochrane/searchHistory?mode=runquery&qnum=19) | | 0 |
| #20 | | ["patient charge"](http://onlinelibrary.wiley.com/o/cochrane/searchHistory?mode=runquery&qnum=20) | | 20 |
| #21 | | ["gatekeeping"](http://onlinelibrary.wiley.com/o/cochrane/searchHistory?mode=runquery&qnum=21) | | 26 |
| #22 | | ["primary health care"](http://onlinelibrary.wiley.com/o/cochrane/searchHistory?mode=runquery&qnum=22) | | 3496 |
| #23 | | [("Health education" OR "patient education" OR "out-of-hours" OR "walk-in centers" OR "continuing care" OR "fast track areas" OR "fast track unit" OR "nurse practitioners" OR "nurse manager" OR "triage" OR "hotline" OR "helpline" OR "telephone consultation" OR " telephone triage" OR "copayment" OR "cost sharing" OR "incentive based" OR "coinsurance" OR "tiered benefit" OR "patient charge" OR "gatekeeping" OR "primary health care")](http://onlinelibrary.wiley.com/o/cochrane/searchHistory?mode=runquery&qnum=23) | | 15620 |
| #24 | | ["emergency medical services"](http://onlinelibrary.wiley.com/o/cochrane/searchHistory?mode=runquery&qnum=24) | | 916 |
| #25 | | ["emergency department"](http://onlinelibrary.wiley.com/o/cochrane/searchHistory?mode=runquery&qnum=25) | | 3388 |
| #26 | | [("emergency medical services" OR "emergency department")](http://onlinelibrary.wiley.com/o/cochrane/searchHistory?mode=runquery&qnum=26) | | 4107 |
| #27 | | ["Cost-benefit analysis"](http://onlinelibrary.wiley.com/o/cochrane/searchHistory?mode=runquery&qnum=27) | | 12751 |
| #28 | | ["cost-effectiveness evaluation"](http://onlinelibrary.wiley.com/o/cochrane/searchHistory?mode=runquery&qnum=28) | | 123 |
| #29 | | ["effectiveness"](http://onlinelibrary.wiley.com/o/cochrane/searchHistory?mode=runquery&qnum=29) | | 56877 |
| #30 | | ["utilization"](http://onlinelibrary.wiley.com/o/cochrane/searchHistory?mode=runquery&qnum=30) | | 8710 |
| #31 | | ["efficacy"](http://onlinelibrary.wiley.com/o/cochrane/searchHistory?mode=runquery&qnum=31) | | 115467 |
| #32 | | ["health care quality"](http://onlinelibrary.wiley.com/o/cochrane/searchHistory?mode=runquery&qnum=32) | | 576 |
| #33 | | ["access"](http://onlinelibrary.wiley.com/o/cochrane/searchHistory?mode=runquery&qnum=33) | | 7090 |
| #34 | | ["length of stay"](http://onlinelibrary.wiley.com/o/cochrane/searchHistory?mode=runquery&qnum=34) | | 8186 |
| #35 | | [" waiting time"](http://onlinelibrary.wiley.com/o/cochrane/searchHistory?mode=runquery&qnum=35) | | 206 |
| #36 | | ["costs"](http://onlinelibrary.wiley.com/o/cochrane/searchHistory?mode=runquery&qnum=36) | | 22921 |
| #37 | | ["Health Services Accessibility"](http://onlinelibrary.wiley.com/o/cochrane/searchHistory?mode=runquery&qnum=37) | | 429 |
| #38 | | [("Cost-benefit analysis" OR "cost-effectiveness evaluation" OR "effectiveness" OR "utilization" OR "efficacy" OR "health care quality" OR "access" OR "length of stay" OR " waiting time" OR "costs" OR "Health Services Accessibility")](http://onlinelibrary.wiley.com/o/cochrane/searchHistory?mode=runquery&qnum=38) | | 75333 |
| #39 | | [("Health education" OR "patient education" OR "out-of-hours" OR "walk-in centers" OR "continuing care" OR "fast track areas" OR "fast track unit" OR "nurse practitioners" OR "nurse manager" OR "triage" OR "hotline" OR "helpline" OR "telephone consultation" OR " telephone triage" OR "copayment" OR "cost sharing" OR "incentive based" OR "coinsurance" OR "tiered benefit" OR "patient charge" OR "gatekeeping" OR "primary health care") AND ("emergency medical services" OR "emergency department") AND ("Cost-benefit analysis" OR "cost-effectiveness evaluation" OR "effectiveness" OR "utilization" OR "efficacy" OR "health care quality" OR "access" OR "length of stay" OR " waiting time" OR "costs" OR "Health Services Accessibility")](http://onlinelibrary.wiley.com/o/cochrane/searchHistory?mode=runquery&qnum=39) | | 426 |
