# Supplementary material for: Effectiveness of Organizational Interventions to Reduce Emergency Department Utilization: A Systematic Review
Source: PLoS One. 2012 May 2;7(5):e35903. doi: 10.1371/journal.pone.0035903 (PMC3342316; doi:10.1371/journal.pone.0035903)
Supplement: Table S2 — Studies examining interventions to improve accessibility of Primary Care (PC). (DOC) [file pone.0035903.s002.doc]

**Table S2.**  Studies examining interventions to improve accessibility of Primary Care (PC).

| **Author; Year [ref]** | **Country** | **Study sample (period of the study; sources of data)** | **Study design** | **Outcomes measures** | **Key findings** | **Quality (0-7)** |
| --- | --- | --- | --- | --- | --- | --- |
| **Increasing PC medical doctors or primary care centers** | | | | | | |
| Sjönell; 1986 [1] | Sweden | 1,441 patients (Survey; 31 December 1979 and 31 December 1981) | Quasi-experimental study with control group | ED visits | Visits to ED were reduced 40% (p<0.001). | 2 |
| Gill; 1996 [2] | USA | 444 clients referred to a PC physician compared with 40,419 clients (Medicaid; medical claims data; 5-months period) | Quasi-experimental study with control group | ED visits | ED visits decreased by 24% in the intervention group and by 4% in the control group. | 2 |
| Bolíbar; 1996 [3] | Spain | 1,974 residents of the city where the PC reforms affected 4 of 7 basic health areas (National Health Service; medical claims data; February 1994) | Cross-sectional | ED visits | Lower percentage of ED use by subjects attending the reorganized centers (27%) than those of non-reorganized (30%) (p>0.05) | 2 |
| Mustard; 1998 [4] | Canada | 657,871 residents of metropolitan Winnipeg (Manitoba Health Services Insurance Plan; Registration files; April 1991 to March 1992) | Cross-sectional | ED visits | Neighbourhoods that had high overall rates of ambulatory care use also had high rates of ED contact. | 1 |
| Bertakis; 1999 [5] | USA | 509 PC patients were randomly assigned to family practice or general internist clinics (No reported; Medical records and interviews) | RCT | ED visits; Hospital admissions | Patients assigned to family practice had significantly fewer ED visits than patients assigned to general internist (*P*=0.02); No differences were found in hospital admissions (*P*=0.81) | 5 |
| Falik; 2001 [6] | USA | 48,738 residents in 24 federally qualified health centers (Medicaid; Medical claims; January to December 1992) | Quasi-experimental study with controls | ED visits; Hospital admissions | Medicaid beneficiaries receiving primary care were less likely to use ED (14.9% vs.15.7%; *P*<0.02) and hospital admission (*P*=0.007) | 2 |
| Stein , 2002 [7] | Brazil | 553 patients (NR; Survey; NR) | Cross-sectional | ED visits | Patients who reported having a primary care physician were more likely to present to the ED appropriately compared with those who stated that they did not have a primary care physician | 2 |
| **Table S2**. Cont. | | | | | | |
| **Author; Year ^ref^** | **Country** | **Study sample (period of the study; sources of data)** | **Study design** | **Outcomes measures** | **Key findings** | **Quality (0-7)** |
| Ionescu-Itu; 2007 [8] | Canada | 95,173 Quebec residents ≥65 years of age (Provincial administrative databases; 2000 and 2001) | Cross-sectional | ED visits | Low continuity in PC was associated with increased ED use (OR 1.25; 95%CI 1.21 to 1.27) | 5 |
| Retchin, 2009 [9] | USA | 18,336 patients 18 years or older at enrolment who had a community primary care physician (Uninsured; Health care records; January 1, 2001, and December 31, 2003) | Quasi-experimental study without control group | ED visits and hospital admissions | There was reduction in the proportion of ED visits (73.9% vs 42.9% *P*<0.001) and hospital admissions (17.6-5 vs 13.8%; *P*<0.001) | 3 |
| Rust, 2009 [10] | USA | All the emergency visits (n=2,070,770) made in the hospital ED (Uninsured; Hospital discharge data; 2003 to 2005) | Case-control study | ED visits | Counties without a community health center primary care clinic site had 33% higher  rates of uninsured all-cause ED visits per 10,000 uninsured population compared with non-CHC  counties (rate ratio=1.33, 95% CI=1.11-1.59) | 5 |
| **Studies examining after-hours primary medical care services interventions** | | | | | | |
| Valdrés-Carroquino; 1993 [11] | Spain | 29,407 patients (National Health Service; Hospital administrative databases; 4^th^ November 1991 to 30^th^ April 1992, and utilizing the facilities over the same period as the previous years) | Quasi-experimental study without control group | ED visits | After the installation of extra-hospital emergency services, ED visits fell from a daily average of 172.6 (SD: 21.3) visits/day to 164.3 (SD: 17.8) visits/day *(P <*0.001) | 1 |
| Alberola-Benavent; 1994 [12] | Spain | 15,290 patients visited in the ED who had different EXTRAHOSPITAL EMERGENCY SERVICES (National Health Service; Hospital administrative database; the first six months of 1992.) | Cross-sectional | ED visits | Visits to the ED were lower in after-hours primary medical care services where the Primary Care Team physically provided ongoing care compared with the other modalities. When Primary Care teams physically provided ongoing care in another town, ED hospital visits increased. | 1 |
| **Table S2**. Cont |  |  |  |  |  |  |
| **Author; Year ^ref^** | **Country** | **Study sample (period of the study; sources of data)** | **Study design** | **Outcomes measures** | **Key findings** | **Quality (0-7)** |
| Chalder; 2003 [13] | UK | 20 ED, 40 general practices, and 14 out-of-hours services within 3 km of a walk-in center or the center of a control town (National Health Service; Each provider consulted; twelve-month periods but the dates not recorded) | Time series | ED visits | A non-significant reduction in ED consultations  (-175 (95%CI -387 to 36) | 3 |
| Chalder; 2003 [13] | UK | 20 ED, 40 general practices, and 14 out-of-hours services within 3 km of a walk-in center or the center of a control town (National Health Service; Each provider consulted; twelve-month periods but the dates not recorded) | Time series | ED visits | A non-significant reduction in ED consultations  (-175 (95%CI -387 to 36) | 3 |
| Hsu; 2003 [14] | UK | Patients of nine general practices of the intervention city and patients of three general practices in the control city (National Health Service; Collection forms and computerized data; January to June 2000 and January to June 2001) | Quasi-experimental study with controls | ED visits | The attendance rate at ED increased by 10% (adjusted rate ratio 1.10, 1.00 to 1.21). | 2 |
| Lowe, 2005 [15] | USA | 57,850 patients assigned to 353 primary care practices (Medicaid; Claims data; August 1,1998 and July 31, 1999) | Cohort study | ED visits | Patients with access to more than 12 evening clinic hours per week used the ED 20% less than patients from practices without evening hours | 2 |
| Van Uden; 2005 [16] | The Netherlands | 2,199 visits before the establishment of the PC physician cooperative, and 2,278 visits after the establishment of cooperative (NR; Registration forms; 3 weeks in 1998 and 3 weeks in 2001) | Quasi-experimental study without control group | ED visits; Mortality | ED visits decreased 53%, but the proportion of patients utilizing primary care increased by 25%; No changes in mortality | 2 |
| **Table S2**. Cont |  |  |  |  |  |  |
| **Author; Year ^ref^** | **Country** | **Study sample (period of the study; sources of data)** | **Study design** | **Outcomes measures** | **Key findings** | **Quality (0-7)** |
| Oterino de la Fuente;  2007 [17] | Spain | All the emergency visits (n=6,454,034) made in the hospital ED and EXTRAHOSPITAL EMERGENCY SERVICES in Asturias (National Health Service; Registration files; 1994 to 2001) | Time series | ED visits | A mean annual increase of 7.8 in the number of emergencies at continuing care points and 5.1% in ED. | 3 |
| Salisbury; 2007 [18] | UK | 400 patients (National Health Service; Direct observations, interviews and administrative medical records; third quarter of 2003 and 2004) | Quasi-experimental study with control group | ED visits | There were no differences between the intervention and control groups in the pre-and post-periods (difference in change 542; 95%CI –347 to 143; *P*=0.23) | 3 |
| Philips, 2010 [19] | Belgium | 5149 patients contacts at ED (Electronic medical records; two months in 2006 , and in 2007) | Quasi-experimental study with control group | ED visits | There was no significant difference in patients between pre- and post-measurement (72% vs 73%). | 5 |
| **Telephone consultation and triage** | | | | | | |
| Darnell; 1985 [20] | USA | 2,627 who had three or more general medicine clinic visits in the previous year (NR; Interviews; NR) | RCT | ED visits | There were no differences between intervention and control groups in ED visits | 3 |
| Lattimer; 1998 [21] | UK | 14,492 calls (National Health Service; 7,308 in the control group and 7184 in the intervention arm) (Database of calls; 23 January 1997 to 20 January 1998) | RCT | ED visits; Mortality | No differences were observed in the number of ED visits and number of deaths. | 3 |
| Lattimer; 2000 [22] | UK | All patients contacting the service during the trial year (National Health Service; University records; January 1997 to January 1998). | RCT | ED admissions within three days after consultation. | 54 fewer adult emergency admissions (11.4 per 1000; *P* = 0.049) within three days of a call. | 6 |
| Vedsted; 2001 [23] | Denmark | All inhabitants of the County of Aarhus (Central database; from 1988 to 1997) | Time series | ED visits | There were no differences in ED visits after telephone triage. | 3 |
| McKinstry; 2002 [24] | UK | 388 patients using the telephone to request same-day appointments (National Health Service; Survey and records; NR) | RCT | Re-visits | Higher re-visits | 6 |
| **Table S2.** Cont. | | | | | | |
| **Author; Year ^ref^** | **Country** | **Study sample (period of the study; sources of data)** | **Study design** | **Outcomes measures** | **Key findings** | **Quality (0-7)** |
| Richards; 2002 [25] | UK | 4,685 patients requesting same-day appointments (National Health Service; Electronic records; One week in every month for 12 months) | Time series | Out-of-hours visits; ED visits; re-visits | More patients used out-of-hours and ED services in the triage group. The mean number of return consultations was greater in triage patients. | 1 |

ED: emergency department; GP: general practitioner; NR: not reported; RCT: randomized controlled trial; Ref: Reference; PC: primary care; OCP: On-going Care Points.

Reference List

1. Sjonell G (1986) Effect of establishing a primary health care centre on the utilization of primary health care and other out-patient care in a Swedish urban area. Fam Pract 3: 148-154.

2. Gill JM, Diamond JJ (1996) Effect of primary care referral on emergency department use: evaluation of a statewide Medicaid program. Fam Med 28: 178-182.

3. Bolibar I, Balanzo X, Armada A, Fernandez JL, Foz G, Sanz E, de la TM (1996) [Impact of the primary health care reform on the use of the hospital emergency services]. Med Clin (Barc ) 107: 289-295.

4. Mustard CA, Kozyrskyj AL, Barer ML, Sheps S (1998) Emergency department use as a component of total ambulatory care: a population perspective. CMAJ 158: 49-55.

5. Bertakis KD, Helms LJ, Azari R, Callahan EJ, Robbins JA, Miller J (1999) Differences between family physicians' and general internists' medical charges. Med Care 37: 78-82.

6. Falik M, Needleman J, Wells BL, Korb J (2001) Ambulatory care sensitive hospitalizations and emergency visits: experiences of Medicaid patients using federally qualified health centers. Med Care 39: 551-561.

7. Stein AT, Harzheim E, Costa M, Busnello E, Rodrigues LC (2002) The relevance of continuity of care: a solution for the chaos in the emergency services. Fam Pract 19: 207-210.

8. Ionescu-Ittu R, McCusker J, Ciampi A, Vadeboncoeur AM, Roberge D, Larouche D, Verdon J, Pineault R (2007) Continuity of primary care and emergency department utilization among elderly people. CMAJ %20;177: 1362-1368.

9. Retchin SM, Garland SL, Anum EA (2009) The transfer of uninsured patients from academic to community primary care settings. Am J Manag Care 15: 245-252.

10. Rust G, Baltrus P, Ye J, Daniels E, Quarshie A, Boumbulian P, Strothers H (2009) Presence of a community health center and uninsured emergency department visit rates in rural counties. J Rural Health 25: 8-16. JRH193 [pii];10.1111/j.1748-0361.2009.00193.x [doi].

11. Valdres CP, citores Augusto JM, Gonzalez EA, Rubio Montaner LI (1993) [The impact on hospital emergency care of the introduction of continuing care into the health centers of Logrono]. Aten Primaria 11: 178-180.

12. Alberola B, V, Rivera CF (1994) [Primary care as a determinant of the utilization of hospital emergency services]. Aten Primaria 14: 825-828.

13. Chalder M, Sharp D, Moore L, Salisbury C (2003) Impact of NHS walk-in centres on the workload of other local healthcare providers: time series analysis. BMJ 326: 532.

14. Hsu RT, Lambert PC, xon-Woods M, Kurinczuk JJ (2003) Effect of NHS walk-in centre on local primary healthcare services: before and after observational study. BMJ 326: 530.

15. Lowe RA, Localio AR, Schwarz DF, Williams S, Tuton LW, Maroney S, Nicklin D, Goldfarb N, Vojta DD, Feldman HI (2005) Association between primary care practice characteristics and emergency department use in a medicaid managed care organization. Med Care 43: 792-800.

16. van Uden CJ, Winkens RA, Wesseling G, Fiolet HF, van Schayck OC, Crebolder HF (2005) The impact of a primary care physician cooperative on the caseload of an emergency department: the Maastricht integrated out-of-hours service. J Gen Intern Med 20: 612-617.

17. Oterino de la FD, Banos Pino JF, Fernandez B, V, Rodriguez-Alvarez A (2007) [Impact in Asturias of primary care emergencies on hospital emergencies for the 1994-2001 period. A time series cointegration analysis]. Rev Esp Salud Publica 81: 191-200.

18. Salisbury C, Hollinghurst S, Montgomery A, Cooke M, Munro J, Sharp D, Chalder M (2007) The impact of co-located NHS walk-in centres on emergency departments. Emerg Med J 24: 265-269.

19. Philips H, Remmen R, Van RP, Teblick M, Geudens L, Bronckaers M, Meeuwis H (2010) What's the effect of the implementation of general practitioner cooperatives on caseload? Prospective intervention study on primary and secondary care. BMC Health Serv Res 10: 222.

20. Darnell JC, Hiner SL, Neill PJ, Mamlin JJ, McDonald CJ, Hui SL, Tierney WM (1985) After-hours telephone access to physicians with access to computerized medical records. Experience in an inner-city general medicine clinic. Med Care 23: 20-26.

21. Lattimer V, George S, Thompson F, Thomas E, Mullee M, Turnbull J, Smith H, Moore M, Bond H, Glasper A (1998) Safety and effectiveness of nurse telephone consultation in out of hours primary care: randomised controlled trial. The South Wiltshire Out of Hours Project (SWOOP) Group. BMJ 317: 1054-1059.

22. Lattimer V, Sassi F, George S, Moore M, Turnbull J, Mullee M, Smith H (2000) Cost analysis of nurse telephone consultation in out of hours primary care: evidence from a randomised controlled trial. BMJ 320: 1053-1057.

23. Vedsted P, Christensen MB (2001) The effect of an out-of-hours reform on attendance at casualty wards. The Danish example. Scand J Prim Health Care 19: 95-98.

24. McKinstry B, Walker J, Campbell C, Heaney D, Wyke S (2002) Telephone consultations to manage requests for same-day appointments: a randomised controlled trial in two practices. Br J Gen Pract 52: 306-310.

25. Richards DA, Meakins J, Tawfik J, Godfrey L, Dutton E, Richardson G, Russell D (2002) Nurse telephone triage for same day appointments in general practice: multiple interrupted time series trial of effect on workload and costs. BMJ 325: 1214.
