# Supplementary material for: Effectiveness of Organizational Interventions to Reduce Emergency Department Utilization: A Systematic Review
Source: PLoS One. 2012 May 2;7(5):e35903. doi: 10.1371/journal.pone.0035903 (PMC3342316; doi:10.1371/journal.pone.0035903)
Supplement: Table S3 — Studies examining educative intervention. (DOC) [file pone.0035903.s003.doc]

**Table S3.** Studies examining educative intervention

| **Author; Year [ref]** | **Country** | **Study sample (Health Care System; period of the study; sources of data)** | **Study design** | **Outcomes measures** | **Key findings** | **Quality**  **(0-7)** |
| --- | --- | --- | --- | --- | --- | --- |
| Rector; 1999 [1] | USA | 6,923 households with a history of ED utilization were randomly assigned to receive a booklet that encouraged use of alternative medicine care (Medicaid; Medical claims data; January 1998-July 1998) | RCT | ED visits | Compared with controls, 1% fewer members of household that receive the booklet visited ED *(P*>0.05) | 5 |
| DeSalvo; 2000 [2] | USA | 288 subjects in the intervention group and 248 subjects in control group (Medicare and Medicaid; Telephonic surveys; NR). | Case-Control study | ED visits | The intervention group had an average of 0.16 ED visits and the control group had 0.29 (*P*<0.01), but at the end of three months the differences were no longer apparent. | 1 |
| Caplan; 2004 [3] | Australia | 739 patients aged 75 and older discharged home from the ED. (NR; Follow-up interviews at 3,6,12 and 18 months by telephone; February 12, 1996 to September 4, 1997) | RCT | ED visits, hospital admissions and mortality | Multidisciplinary intervention was not associated with differences in ED visits (15.7% intervention group versus 13.3% control group; *P*=0.35), or on mortality rates. Intervention patients had a lower rate of all admissions to the hospital (*P*=0.48) | 6 |
| Scott; 2004 [4] | USA | 294 adults, aged 60 and older, with 11 or more outpatient visits in the prior 18 months and/or one or more self-reported chronic conditions (HMO; Administrative databases; February 1995 to July of 1996) | RCT | ED visit hospital admissions and mortality | Intervention group had fewer ED visits (*P*=0.008) and hospital admissions (*P*=0.12). Mortality did not differ between groups. | 5 |
| **Table S3 cont.** |  |  |  |  |  |  |
| **Author; Year [ref]** | **Country** | **Study sample (Health Care System; period of the study; sources of data)** | **Study design** | **Outcomes measures** | **Key findings** | **Quality**  **(0-7)** |
| Michelen, 2006 [5] | USA | 711 patients (Northern Manhattan Community Voices; Survey Form and ED records; January 2003 to December 2004) | Quasi-experimental study without control group | ED visits | Educational intervention were significantly correlated with decreased ED visits *(P <*0.001) | 3 |
| Bird; 2007 [6] | Australia | 231 patients over 55 years who made three or more visits at ED in the previous 12 months (Regularly interview; February 2004 and 1 October 2005) | Quasi-experimental study with control group | ED visits, hospital admissions | 20.8% reduction in ED visits in the intervention group, and a 19.2% reduction in hospital admissions. | 4 |

ED: emergency department; GP: general practitioner; NR: not reported; RCT: randomized controlled trial; Ref: reference; PC: primary care

Reference List

1. Rector TS, Venus PJ, Laine AJ (1999) Impact of mailing information about nonurgent care on emergency department visits by Medicaid beneficiaries enrolled in managed care. Am J Manag Care 5: 1505-1512.

2. DeSalvo A, Rest SB, Nettleman M, Freer S, Knight T (2000) Patient education and emergency room visits. Clin Perform Qual Health Care 8: 35-37.

3. Caplan GA, Williams AJ, Daly B, Abraham K (2004) A randomized, controlled trial of comprehensive geriatric assessment and multidisciplinary intervention after discharge of elderly from the emergency department--the DEED II study. J Am Geriatr Soc 52: 1417-1423.

4. Scott JC, Conner DA, Venohr I, Gade G, McKenzie M, Kramer AM, Bryant L, Beck A (2004) Effectiveness of a group outpatient visit model for chronically ill older health maintenance organization members: a 2-year randomized trial of the cooperative health care clinic. J Am Geriatr Soc 52: 1463-1470.

5. Michelen W, Martinez J, Lee A, Wheeler DP (2006) Reducing frequent flyer emergency department visits. J Health Care Poor Underserved 17: 59-69.

6. Bird SR, Kurowski W, Dickman GK, Kronborg I (2007) Integrated care facilitation for older patients with complex health care needs reduces hospital demand. Aust Health Rev 31: 451-461.
