# Supplementary material for: Effectiveness of Organizational Interventions to Reduce Emergency Department Utilization: A Systematic Review
Source: PLoS One. 2012 May 2;7(5):e35903. doi: 10.1371/journal.pone.0035903 (PMC3342316; doi:10.1371/journal.pone.0035903)
Supplement: Table S4 — Studies examining barriers interventions. (DOC) [file pone.0035903.s004.doc]

**Table S4.** Studies examining barriers interventions

| **Author; Year [ref]** | **Country** | **Study sample (period of the study; sources of data)** | **Study design** | **Outcomes** | **Key findings** | **Quality**  **(0-7)** |
| --- | --- | --- | --- | --- | --- | --- |
| **Cost-sharing** | | | | | | |
| O’Grady; 1985 [1] | USA | 3,973 persons <62 years of age (Insurance claim forms completed by providers; NR) | RCT | ED visits | 42% increase in ED visits in persons with no cost sharing when compared with persons who had 95 per cent payment plan (*P*<0.01) | 6 |
| Selby; 1996 [2] | USA | 30,276 subjects, age range 1 to 63 years at the start of the study (Kaiser Permanente HMO in northern California; Registration data base; 1992 to 1993) | Prospective cohort study | ED visits; Hospital admissions; Mortality | 15% decrease in ED visits with the introduction of a small co-payment. No increase in mortality or in the number of potentially avoidable hospitalization | 3 |
| Murphy; 1997 [3] | Ireland | 88504 participants (General Medical Service ineligibles; Hospital database; One year before and one year after the implementation of the new regulation on 1 March of 1994) | Quasi-experimental study without control group | ED visits | The overall workload of the ED was unaffected, but with a slightly significant reduction in the number of patients who attended with non-emergency pathologies | 1 |
| Wong; 2001 [4] | USA | 1,700 chronically ill patients who completed the 12 and 18 months surveys (NR; Surveys; Two weeks in February and October 1986) | Prospective Cohorts | ED visits | 60% decrease in visits for minor symptoms in high co-pay group compared with no co-pay group; also showed a 78% decrease in visits for serious symptoms in high co-pay group. | 2 |
| Hsu; 2004 [5] | USA | 695 patients in an integrated delivery system (Kaiser Permanente-Northern California; Survey; October 2001) | Cross-sectional | ED visits | 11.3% patients who reported having a co-payment stated that they delayed or avoided emergency care. | 3 |
| Reed; 2005 [6] | USA | 932 patients with cost-sharing (Kaiser Permanente-Northern California;Telephone interviews, January 2000) | Cross-sectional | ED visits | 19% patients reported changing their care-seeking behavior, 9% delayed going to ED, and 2% avoided medical care. 41% of the subjects correctly reported the amount of the copayment | 2 |
| Hsu; 2006 [7] | USA | 2,257,445 commercially insured and 261,091 Medicare insured health-system members and 59,557 contemporaneous controls (Kaiser Permanente-Northern California database; January 1999 through December 2001) | Quasi-experimental longitudinal study with controls | ED visits; adverse outcomes. | 23% decrease in ED visits with the $50-100 co-payment and 12% decrease with $20-30 co-payment. Hospitalizations, ICU admissions, and death did not increase with co-payment | 5 |
| **Table S4**. Cont. | | | | | | |
| **Author; Year [ref]** | **Country** | **Study sample (period of the study; sources of data)** | **Study design** | **Outcomes** | **Key findings** | **Quality**  **(0-7)** |
| Wharam; 2007 [8] | USA | 8,724 persons aged between 1 and 64 years and insured by a Massachusetts health plan (High-deductible plan vs HMO; Claims database; March 1, 2001, and June 30, 2005 | Quasi-experimental longitudinal study with controls | ED visits; hospital admissions | 10% decrease in ED visits in HDHP group compared with control group. High-deductible coverage had decrease in the rate of hospitalization. | 4 |
| Hartung; 2008 [9] | USA | 116,822 individuals affected by the co-pay policy (Medicaid; Medical claims; 36 months periods) | Quasi-experimental longitudinal study with controls | ED visits | No changes in ED visits | 4 |
| Wilson; 2008 [10] | USA | 121,098 enrolees in a consumer-driven health plan and 612,954 enrolees in a comprehensive major medical (Blue Cross and Blue Shield of Minnesota; Medical claims; 2004, 2005 and 2006 years) | Quasi-experimental longitudinal study with controls | ED visits; Hospital admissions | 11.2% decrease in ED visits and 17% decrease in hospital admissions from patients enrolled in a consumer deductible health-care compared to those enrolled in a comprehensive major medical care plan | 2 |
| Lowe, 2010 [11] | USA | 414,009 adults (Oregon Health Plan enrolees; claims data; Three periods; January 1, 2001 through Februray 29, 2003; March 1, 2003, through May 31, 2004; and June 1, 2004, through December 31, 2004) | Quasi-experimental study with control group | ED visits | Compared with the control group, adjusted case-mix ED utilization rates fell 18%. | 4 |
| Waters 2011[12] | USA | 1376 participants who were initially enrolled in a PPO plan in 2005, and switched to high-deductible health plan. Control group was a pool enrolled in PPO plan for all study (Blue Cross and Blue Shield of Minnesota; 2005 through 2007) | Quasi-experimental study with control group | ED visits | High-deductible health plan enrolment was associated with reduced ED visits (*P*<0.001) | 4 |
| **Gate-keeping** | | | | | | |
| Hurley; 1989 [13] | USA | 3,000 patients enrolled in 4 programs that used a gatekeeper model and compared with non-enrolled patients (Medicaid; Administrative data claims; NR) | Cross-sectional | ED visits | The gate-keeping plans were successful in reducing ED use for enrolees compared to the comparison group. | 2 |
| **Table S4**. Cont. | | | | | | |
| **Author; Year [ref]** | **Country** | **Study sample (period of the study; sources of data)** | **Study design** | **Outcomes** | **Key findings** | **Quality**  **(0-7)** |
| Young; 1997 [14] | USA | 980 ED visits with managed care insurance to document gate-keeping interactions (Manage Care Organization; Administrative data; last 6 months of 1995 and the first 6 months of 1996) | Prospective cohort | Adverse outcomes | 20.2% were denied attention and 13.8% had adverse outcomes. | 2 |
| Derlet; 1997 [15] | USA | 516 patients denied authorization for ED care | Prospective cohorts | Re-visits; adverse outcomes | We found that 516 patients denied authorization, 9 later return to an ED with serious medical condition | 2 |
| Viner; 2000 [16] | USA | 151 patients did not receive authorization for ED care (Managed Care Organization insurance; Telephone survey; November 1996, through May 30,1997) | Prospective cohorts | Re-visits; adverse outcomes | 151 patients did not receive MCO authorization for ED care. A significant number of patients (11%) return visits to the ED with an admission rate of 4%. | 2 |
| Schillinger, 2000 [17] | USA | 2,293 patients of 28 PC physicians (Hospital’s information system; April 1, 1997 to April 1, 1998) | RCT | ED visits; Hospital admissions | No changes in ED visits (0.06, 95%CI -0.09 to 0.22). Intervention patients decreased yearly hospitalizations by 0.14 visits per year more than control patients (*P*=0.02) | 5 |

ED: emergency department; GP: general practitioner; NR: not reported; RCT: randomized controlled trial; Ref: reference; PC: primary care

Reference List

1. O'Grady KF, Manning WG, Newhouse JP, Brook RH (1985) The impact of cost sharing on emergency department use. N Engl J Med 313: 484-490.

2. Selby JV, Fireman BH, Swain BE (1996) Effect of a copayment on use of the emergency department in a health maintenance organization. N Engl J Med 334: 635-641.

3. Murphy AW, Leonard C, Plunkett PK, Bury G, Lynam F, Smith M, Gibney D (1997) Effect of the introduction of a financial incentive for fee-paying A&E attenders to consult their general practitioner before attending the A&E department. Fam Pract 14: 407-410.

4. Wong MD, Andersen R, Sherbourne CD, Hays RD, Shapiro MF (2001) Effects of cost sharing on care seeking and health status: results from the Medical Outcomes Study. Am J Public Health 91: 1889-1894.

5. Hsu J, Reed M, Brand R, Fireman B, Newhouse JP, Selby JV (2004) Cost-sharing: patient knowledge and effects on seeking emergency department care. Med Care 42: 290-296.

6. Reed M, Fung V, Brand R, Fireman B, Newhouse JP, Selby JV, Hsu J (2005) Care-seeking behavior in response to emergency department copayments. Med Care 43: 810-816.

7. Hsu J, Price M, Brand R, Ray GT, Fireman B, Newhouse JP, Selby JV (2006) Cost-sharing for emergency care and unfavorable clinical events: findings from the safety and financial ramifications of ED copayments study. Health Serv Res 41: 1801-1820.

8. Wharam JF, Landon BE, Galbraith AA, Kleinman KP, Soumerai SB, Ross-Degnan D (2007) Emergency department use and subsequent hospitalizations among members of a high-deductible health plan. JAMA 297: 1093-1102.

9. Hartung DM, Carlson MJ, Kraemer DF, Haxby DG, Ketchum KL, Greenlick MR (2008) Impact of a Medicaid copayment policy on prescription drug and health services utilization in a fee-for-service Medicaid population. Med Care 46: 565-572.

10. Wilson AR, Bargman EP, Pederson D, Wilson A, Garrett NA, Plocher DW, Ailiff PL, Jr. (2008) More preventive care, and fewer emergency room visits and prescription drugs--health care utilization in a consumer-driven health plan. Benefits Q 24: 46-54.

11. Lowe RA, Fu R, Gallia CA (2010) Impact of policy changes on emergency department use by Medicaid enrollees in Oregon. Med Care 48: 619-627.

12. Waters TM, Chang CF, Cecil WT, Kasteridis P, Mirvis D (2011) Impact of high-deductible health plans on health care utilization and costs. Health Serv Res 46: 155-172. 10.1111/j.1475-6773.2010.01191.x [doi].

13. Hurley RE, Freund DA, Taylor DE (1989) Gatekeeping the emergency department: impact of a Medicaid primary care case management program. Health Care Manage Rev 14: 63-71.

14. Young GP, Lowe RA (1997) Adverse outcomes of managed care gatekeeping. Acad Emerg Med 4: 1129-1136.

15. Derlet RW, Young GP (1997) Managed care and emergency medicine: conflicts, federal law, and California legislation. Ann Emerg Med 30: 292-300.

16. Viner KM, Bellino M, Kirsch TD, Kivela P, Silva JC (2000) Managed care organization authorization denials: lack of patient knowledge and timely alternative ambulatory care. Ann Emerg Med 35: 272-276.

17. Schillinger D, Bibbins-Domingo K, Vranizan K, Bacchetti P, Luce JM, Bindman AB (2000) Effects of primary care coordination on public hospital patients. J Gen Intern Med 15: 329-336.
